# Near-Infrared Imaging of Adoptive Immune Cell Therapy in Breast Cancer Model Using Cell Membrane Labeling

**DOI:** 10.1371/journal.pone.0109162

**Published:** 2014-10-21

**Authors:** Fatma M. Youniss, Gobalakrishnan Sundaresan, Laura J. Graham, Li Wang, Collin R. Berry, Gajanan K. Dewkar, Purnima Jose, Harry D. Bear, Jamal Zweit

**Affiliations:** 1 Department of Radiology, Center for Molecular Imaging, Virginia Commonwealth University, Richmond, Virginia, United States of America; 2 Department of Surgery, Division of Surgical Oncology, Virginia Commonwealth University, Richmond, Virginia, United States of America; 3 Massey Cancer Center, Virginia Commonwealth University, Richmond, Virginia, United States of America; Carl-Gustav Carus Technical University-Dresden, Germany

## Abstract

The overall objective of this study is to non-invasively image and assess tumor targeting and retention of directly labeled T-lymphocytes following their adoptive transfer in mice. T-lymphocytes obtained from draining lymph nodes of 4T1 (murine breast cancer cell) sensitized BALB/C mice were activated in-vitro with Bryostatin/Ionomycin for 18 hours, and were grown in the presence of Interleukin-2 for 6 days. T-lymphocytes were then directly labeled with 1,1-dioctadecyltetramethyl indotricarbocyanine Iodide (DiR), a lipophilic near infrared fluorescent dye that labels the cell membrane. Assays for viability, proliferation, and function of labeled T-lymphocytes showed that they were unaffected by DiR labeling. The DiR labeled cells were injected via tail vein in mice bearing 4T1 tumors in the flank. In some cases labeled 4T1 specific T-lymphocytes were injected a week before 4T1 tumor cell implantation. Multi-spectral *in vivo* fluorescence imaging was done to subtract the autofluorescence and isolate the near infrared signal carried by the T-lymphocytes. In recipient mice with established 4T1 tumors, labeled 4T1 specific T-lymphocytes showed marked tumor retention, which peaked 6 days post infusion and persisted at the tumor site for up to 3 weeks. When 4T1 tumor cells were implanted 1-week post-infusion of labeled T-lymphocytes, T-lymphocytes responded to the immunologic challenge and accumulated at the site of 4T1 cell implantation within two hours and the signal persisted for 2 more weeks. Tumor accumulation of labeled 4T1 specific T-lymphocytes was absent in mice bearing Meth A sarcoma tumors. When lysate of 4T1 specific labeled T-lymphocytes was injected into 4T1 tumor bearing mice the near infrared signal was not detected at the tumor site. In conclusion, our validated results confirm that the near infrared signal detected at the tumor site represents the DiR labeled 4T1 specific viable T-lymphocytes and their response to immunologic challenge can be imaged *in vivo*.

## Introduction

Cancer treatment by adoptive immune cell therapy (AIT) is a form of immunotherapy that relies on the *in vitro* activation and/or expansion of immune cells. In this approach, immune cells, particularly CD8+ T lymphocytes, can potentially be harvested from a tumor-bearing patient, then activated and/or expanded *in vitro* in the presence of cytokines and other growth factors, and then transferred back into the same patient to induce tumor regression. AIT allows the *in vitro* generation and activation of T-lymphocytes away from the immunosuppressive tumor microenvironment, thereby providing optimum conditions for potent anti-tumor activity. Dudley and colleagues [1], demonstrated partial and complete responses in about 50% of patients with metastatic melanoma following adoptive transfer of *ex vivo*-expanded autologous tumor-infiltrating T lymphocytes. Another example of AIT success was illustrated by Yee and colleagues [2], in the treatment of melanoma patients. Their results showed that adoptively transferred T-cell clones persist *in vivo* and preferentially localize to tumor sites and mediate an antigen-specific immune response characterized by metastases regression in 8 of 10 patients. Another study showed that the re-infusion of engineered lymphocytes to express melanoma-specific T cell receptor (TCR) induced regression in 13% of patients [3]. The success of AIT relies on the *ex vivo* manipulation of tumor specific T-lymphocytes to produce a large number of cells with optimized anti-tumor effector functions [4]. We have previously shown that *in vitro* treatment of tumor antigen sensitized draining lymph node (DLN) cells with bryostatin and ionomycin (B/I) selectively activates antigen-sensitized T lymphocytes that can induce regression of several different murine tumors, either primary or metastatic, and can confer long-term resistance to re-challenge [5]–[7]. Several groups are also investigating and exploring the possibility of using cocktail of growth factors during *ex vivo* expansion of T-lymphocytes. However, during the development of such methodologies it is often difficult to evaluate the functioning and potency of the adoptively transferred T-cells *in vivo*. Tracking the movement, proliferation and viability of re-infused tumor specific T-lymphocytes, by multi-modality imaging in serial images at different time points, during the treatment could define the parameters that lead to successful AIT. To address these issues, various non-invasive imaging methods to monitor immune cell trafficking and activation are under development in various laboratories [8]–[11]. Herein, we describe a simple near infra-red (NIR) optical imaging method that allowed us to monitor the tumor homing of adoptively transferred T-cells in an animal model of breast cancer.

Noninvasive *in vivo* imaging of adoptively transferred T-lymphocytes requires an effective and mild labeling methodology and the right imaging technology. Cell labeling methods for imaging purposes can be classified as either direct or indirect [8]. Direct labeling enables the imaging of cells labeled *ex vivo* and reinfused into the patient or subject who is being examined. Radionuclide based direct labeling probes for SPECT/PET imaging includes include, [^111^In]-Oxine, [^18^F-]FDG, [^99m^Tc]-HMPAO, and [^64^Cu]-PTSM. These have been used to monitor the migration of T-lymphocytes *in vivo* [12]–[17]. However, these techniques do not permit long term monitoring due to the short physical half-life of the radionuclides, relatively low level of radioactivity per cell, and in certain instances, are limited by significant cell toxicity [18]. On the other hand, indirect labeling utilizes imageable reporter protein/probe combinations and rely on the *ex vivo* transduction of a relevant reporter gene into the immune-competent cells. Following reinfusion of the transduced cells into the subject, the product of the reporter gene can be imaged by injecting a suitable radiolabeled substrate. Herpes Simplex Virus Type-1 Thymidine Kinase (HSV1-tk) is such a PET reporter gene (PRG) and is one of the most commonly used indirect methods to image adoptively transferred T-lymphocytes in animals and patients, and it can be imaged with various substrates such as[^124^I]-FIAU, [^131^I]-FIAU, [^18^F]-FEAU and [^18^F]-FHBG [19]–[26]. The sodium/iodide symporter (NIS) is another reporter gene that has also been evaluated for imaging cells of the immune system in immortalized macrophage cell lines genetically engineered to express NIS and GFP (RAW264.7/hNIS-GFP) [27]. Using [^124^I-NaI PET imaging, these investigators monitored macrophage migration towards inflamed tissue. In addition to [^124^I]-NaI; [^99m^Tc]-0_4_, [^123^I]-NaI, [^125^I]-NaI and [^18^F]-tetrafluoroborate can also be used as substrates for NIS [28].

Radionuclide based methods are useful in the clinic due to the ability to image signals from deep tissues. However, the logistics of radionuclide techniques are more demanding, which makes them less cost effective for preclinical investigations. Non-radioactive agents, such as fluorocarbon-based probes, have also been used for efficient direct *ex vivo* labeling of cells, and used in conjunction with ^19^F MRI for *in vivo* detection and imaging [29]. This method, for *in vivo* monitoring of an autologous dendritic cell vaccine to treat colorectal cancer, is currently being evaluated in humans [30].

On the other hand, optical methods are relatively easy, require simpler imaging equipment and are cost effective, but are not very useful in the clinic due to the lack of effective technology that can detect optical signal from deep tissues. However, optical labels, both direct and indirect, could be very useful for research and development of various strategies aimed at improving immune cell therapy. One cost effective approach is to use optical reporter proteins such as firefly or renilla luciferase to label the cells and monitor them by optical imaging [31], [32]. However, these are currently limited to animal models and their success relies on the successful stable transduction of the reporter gene and the persistence of the reporter gene expression over time. A combination of direct labeling strategy and optical imaging methodology would be ideal, in terms of simplicity and cost effectiveness, for monitoring cell trafficking in preclinical studies. This could be extended to clinical investigations when a suitable imaging nuclide, such as ^124^I or ^19^F, is included in the direct cell labeling strategy.

Studies describing optical imaging of directly labeled immune cells is quite limited [8]. However, using *ex vivo* imaging and other *in vitro* protocols, several DNA-binding, cytoplasmic, covalent coupling or membrane inserting fluorescent dyes have been investigated for direct labeling of T lymphocytes to track lymphocyte migration and proliferation [33]. Due to interference from autofluorescence and tissue absorbance, most of these dyes are not ideal for *in vivo* optical imaging and their use is limited to *ex vivo* detection. The optical imaging window is primarily limited by absorption, due to either blood at short wavelengths or water at long wavelengths. This could be overcome by the use of near-infrared dyes (NIR) that will allow deep tissue signal localization [34]. The red/NIR tissue optical window between 600 and 1300 nm is the spectral region where light has its maximum depth of penetration in tissues due to minimal absorption and scattering. Among the various options available for direct labeling, the cell membrane dyes retain the signal for relatively longer periods than DNA-binding, cytoplasmic or covalent coupling dyes [33]. Therefore, a combination of NIR based fluorescent cell membrane dyes would offer a direct labeling method for monitoring cell trafficking while exploiting high sensitivity, simple labeling technique, decreased autofluorescence and relatively low costs [18].

In this study, using a lipophilic, NIR-fluorescent cyanine cell membrane dye and a direct labeling method, we investigated the tumor localization and migration of 4T1 tumor specific T-lymphocytes that were first activated *in vitro* with Bryostatin/Ionomycin (B/I) and grown with Interleukin-2 (IL-2). We were able to monitor up to three weeks post intravenous administration of directly labeled T-lymphocytes in living animals by non-invasive multispectral fluorescence imaging.

## Materials and Methods

### Animals

Female BALB/c mice, aged between 8–12 weeks, obtained from National Cancer Institute, Bethesda, MD, were caged in groups of five or fewer, and provided with food and water ad libitum. All animal experiments were performed according to the policies and guidelines of the Institutional Animal Care and Use Committee (IACUC) at Virginia Commonwealth University, USA. All procedures were reviewed and approved by the VCU IACUC prior to the conduct of the study. 2% Isoflurane in Oxygen was used for anesthesia during imaging and all efforts were made to minimize suffering. Animals were euthanized by using controlled release of CO_2_ from a cylinder into a chamber that allows good visibility of the animals and large enough to avoid crowding.

### Tumor cell lines

Murine mammary breast carcinoma (4T1) cell line was kindly provided by Dr. Jane Tsai, Michigan Cancer Foundation, Detroit, Michigan [35]. 4T1 cells were maintained in Dulbecco's Modified Eagle Medium (DMEM) with 10% heat-inactivated fetal bovine serum (Hyclone, Logan, UT) 1 mM sodium pyruvate, 100 U/ml penicillin, and 100 µg/ml streptomycin (Sigma, St. Louis MO). Tumor cells were trypsinized with 0.05% trypsin-EDTA (Fisher, Pittsburgh), washed with PBS and used for experiments.

Meth A sarcoma cells were obtained from American Type Culture Collection (Rockville, MD) and grown in DMEM media, containing 10% heat inactivated fetal bovine serum, 1 M sodium pyruvate, 100 U/ml penicillin, and 100 µg/ml streptomycin, 10 mM HEPES, and 2 mM L-glutamine in a humidified incubator with 5% CO_2_, at 37°C. Cells >90% confluence were used for experiments.

### Isolation, activation and in vitro expansion of tumor specific lymphocytes

Donor mice were vaccinated in the left hind footpad with viable 1×10^6^ 4T1 cells. Ten days later, when these mice had a growing tumor in the foot, popliteal draining lymph nodes (DLN) were harvested under sterile conditions and disrupted through mesh screens to yield single cell suspensions. The cells from DLN were filtered and resuspended in complete RPMI media at 1×10^6^ cell/ml concentration. These cells were then activated by incubating them with 5 nM Bryostatin 1 (Sigma, St. Louis MO), 1 µM Ionomycin (Calbiochem, San Diego, CA) and 80 U/ml IL-2 (Chiron, Emeryville, CA) at 37°C for 18 hours. To expand the lymphocyte population, cells were washed three times with warm, complete RPMI and resuspended at 1×10^6^ cells/ml concentration and incubated with 40 U/ml of IL-2. The cells were then allowed to proliferate in culture for 6–13 days and were split every other day to maintain 1–2×10^6^ cells/ml concentration. Fresh IL-2 was added at each split. Cell proliferation was calculated by determining the number of viable cells, on different days of culture, by trypan blue exclusion and comparing it with the number of cells on day 1 of expansion. Luminescence based cell viability assays were also carried out as described below.

### Labeling of lymphocytes with NIR-fluorescent probe

Direct labeling of T- lymphocytes isolated on day-6 after *in vitro* expansion was achieved by incubating the cells with 1,1-dioctadecyltetramethyl indotricarbocyanine Iodide (DiIC18(7) or DiR), a lipophilic Near-Infra red fluorescent dye (Absorption/Emission: 748/780 nm) (PerkinElmer, MA). DiR stock solution was prepared by dissolving 25 mg dye in 3 ml Ethanol. From this stock, further dilutions were made to required concentrations in media and incubated with cells for 30 minutes at 37°C. After incubation, cells were spun down at 1000 rpm at 4°C for 10 minutes, then washed twice with PBS and used for experiments.

### Cell viability assays

The highest tolerable concentration of DiR in the staining solution, which can be used to label T-cells, was determined using Cell Titer-Glo Luminescent assay kit (Promega, USA) and flow cytometry. Tumor sensitized lymphocytes, cultured in media for 6 days in IL-2 containing media, were labeled with a staining solution containing various concentrations (3.5, 14, 56, 224 and 320 µg/ml) of DiR. Following labeling, 25000 cells/100 µl were plated in triplicates in a 96 well plates, and incubated along with similar number of unlabeled cells in a humidified 37°C CO_2_ incubator. After 1, 4 or 7 days of incubation, the Cell Titer-Glo reagent was added to the cells and the % Viability calculated for each labeled group in comparison to unlabeled cells (100% viability). For flow cytometry, DiR labeled and unlabeled T-cells (2 or 7 days post labeling) were double stained with propidium iodide (PI) and AnnexinV-FITC (BD Biosciences Pharmingen, San Diego, CA) and analyzed using Beckman Coulter Epics XL-MCL flowcytometer and the data were analyzed using EXPO 32 software. Two different sets of experiments were performed to verify the results.

### Interferon-γ release assay

#### *In vitro*

Interferon-γ (IFN- γ) released into supernatants of 4T1 sensitized and expanded T-lymphocytes in response to stimulation with irradiated 4T1 and Meth-A cells (non-specific control) was assayed using BD OptEIA mouse IFN-γ ELISA kit (BD Biosciences, San Jose, CA). T-cells obtained from popliteal DLN, which had been activated and expanded in IL-2 containing media, were collected on day 6 and incubated with DiR staining solution (320 µg/ml). The DiR labeled and unlabeled cells were collected 1 day and 7 days post-labeling and incubated with the irradiated 4T1 or Meth-A tumor cells for 24 hrs, and the amount of IFN-γ in the supernatant was analyzed. As a negative control, 4T1 sensitized T cells alone were incubated under similar conditions.

#### *In vivo*

Blood was collected from three different groups of animals bearing: i) 10 day old 4T1 tumor, ii) 10 day old 4T1 tumor and injected with 10 million DiR labeled T-cells/mouse, iii) 10 day old Meth-A tumor and injected with similar number of activated and DiR labeled T-cells. One week later, blood was collected and the amount of the interferon-γ in serum was determined by the same kit and assay described above.

### Animal model and lymphocyte trafficking

For the development of tumor models, mice were inoculated subcutaneously into the right flank with either 4T1 cells or Meth-A tumor cells (5×10^4^) and the tumor was allowed to grow for 10 days. To follow *in vivo* trafficking of T cells, 5×10^6^ 4T1 sensitized T-cells were first labeled with DiR (320 µg/ml staining solution). The cells were then filtered through 70-µm nylon mesh strainer and injected intravenously (iv) into the mouse tail vein (10×10^6^ cells in 0.5 ml PBS per mouse) either a week before the tumor cells were injected or 10 days after tumor implantation. One day before injection of DiR labeled T-lymphocytes, mice were pretreated, intraperitoneally (ip), with 100 mg/kg Cyclophosphamide (CYP) (Mead Johnson, Princeton, NJ). In order to understand the fate of non-viable DiR labeled cells, parallel group of 4T1 tumor bearing mice were injected with cell lysate derived from the DiR labeled T-lymphocytes.

### Multi-spectral fluorescence imaging

Fluorescent images were taken from day 1 to day 21 to monitor the homing and localization of labeled T lymphocytes at the tumor site. Multi-spectral fluorescence imaging system (Maestro-2 by PerkinElmer, USA) was used to monitor DiR labeled T-lymphocyte trafficking and localization in host mice with and without tumors. A NIR/Orange double filter setup (640 nm to 820 nm) and spectral unmixing was used for image acquisition and processing. Image processing and data analysis were performed using Maestro-2 software version 2.10. Images were obtained at different time points (2 hours, 24 hours, 48 hours, 72 hours, 6-days, 8-days, 10-days, 13-days, 15-days, 17-days, 20-days, and 21-days) post-injection of labeled T-lymphocytes. Six mice per time point were used; and four imaging positions, dorsal, ventral, left and right sides, were used at each time point.

### Immunohistochemistry

After imaging, mice were euthanized; organs and tumors were collected, embedded in Optimal Cutting Temperature compound (O.C.T, Tissue-Tek, USA) and stored at −80°C. Using a cryostat system (LEICA CM1850 UV), five-micron thick serial sections of the tissue samples were collected onto glass slides. The sections were fixed at room temperature for 15 min in 3% freshly prepared paraformaldehyde in PBS (pH 7.4), and then washed three times with 0.3M glycine in PBS. Antigen retrieval was done using heat and citric acid buffer method. Following this, sections were washed three times in PBS for 5 min each, then incubated with blocking buffer (5% normal goat serum +0.1% Triton X-100 in PBS, pH 7.4) for 1 hour at room temperature. Tissue sections were incubated, overnight, at 4°C with Anti-F4/80 and Anti-CD69 primary antibodies (Abcam, USA), which were used to detect macrophages and activated T cells respectively. This was followed by incubation, for 1 hr at room temperature, with Alexa Fluor 488 goat anti-rat IgG and Alexa Fluor 568 Goat anti-hamster IgG (Abcam, USA). The sections were cover-slipped and mounted with Vectashield Mounting Medium (Vector Laboratories, Burlingame, CA), and were then imaged and analyzed using a Zeiss LSM 700 confocal laser scanning microscope.

### Statistical analysis

The significant differences in the *ex vivo* and *in vivo* studies were determined using the equal variance analysis (ANOVA) (JMP software), or Student's t-test. P value of <0.05 was considered as statistically significant difference. The data shown in graphs are mean ± SEM.

## Results

### Viability of DiR labeled T lymphocytes

**Figure 1:**
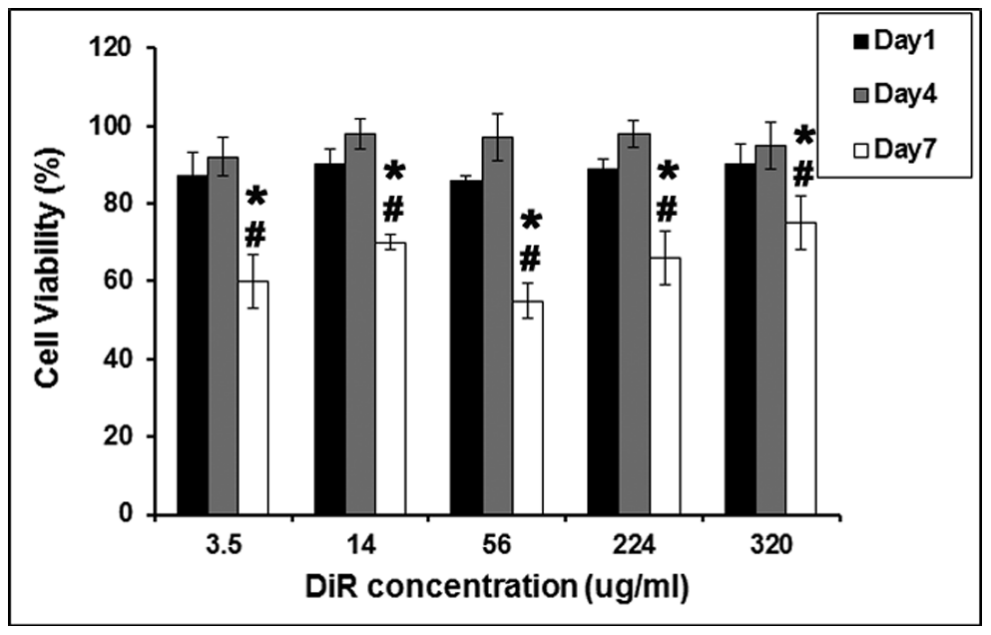
Percent viability of T cells labeled with various concentrations of DiR measured using ATP based luminescent assay. Bryostatin/Ionomycin (B/I) activated 4T1-cells grown in IL-2 were labeled on day 6 of their ex vivo expansion, which is day-0 of labeling. Viability of T-cells on 1, 4 and 7 days post-labeling were compared with unlabeled cells on respective days and expressed as percent viability. T-test was used to compare the significant differences (p<0.05) in the cell viability between day 1 versus day 7 (*) and day 4 versus day 7 (#). Average ± SD from three different assays are shown in the graph.

The viability of lymphocytes labeled with various concentrations of DiR was assessed to determine the optimum non-toxic concentration with which cells could be labeled. Since T cells, which have been B/I activated and expanded in IL-2 containing media for six days, were used for *in vivo* studies, the viability of cells from day 6 onwards was monitored, when labeled with DiR and compared with unlabeled T cells. The results show that there are no significant differences in % Viability among the various concentrations of DiR tested. However there is a significant difference in the viability of cells recovered on days 1 and 4 compared to viability tested at day 7 post-labeling. Since on day 1 and 4 the cells were 80–90% viable even at the highest concentration of DiR used (320 µg/ml), this concentration was used for further *in vitro* and *in vivo* studies (Figure 1) as higher amount of DiR on cell membranes is likely to allow signal retention for relatively longer durations. Flow cytometry analysis also confirmed that, at this concentration the efficiency of labeling was >90% (Figure S1 in File S1). Tumor sensitized lymphocytes were labeled with 320 µg/ml of DiR and on day 2 and 7 post-labeling, cells were taken and incubated with Annexin V/PI to determine the cause of cell death. The flow cytometry results showed that, the cell viability of unlabeled and labeled cells was comparable.

### Pre- and post-labeling Cell proliferation

**Figure 2:**
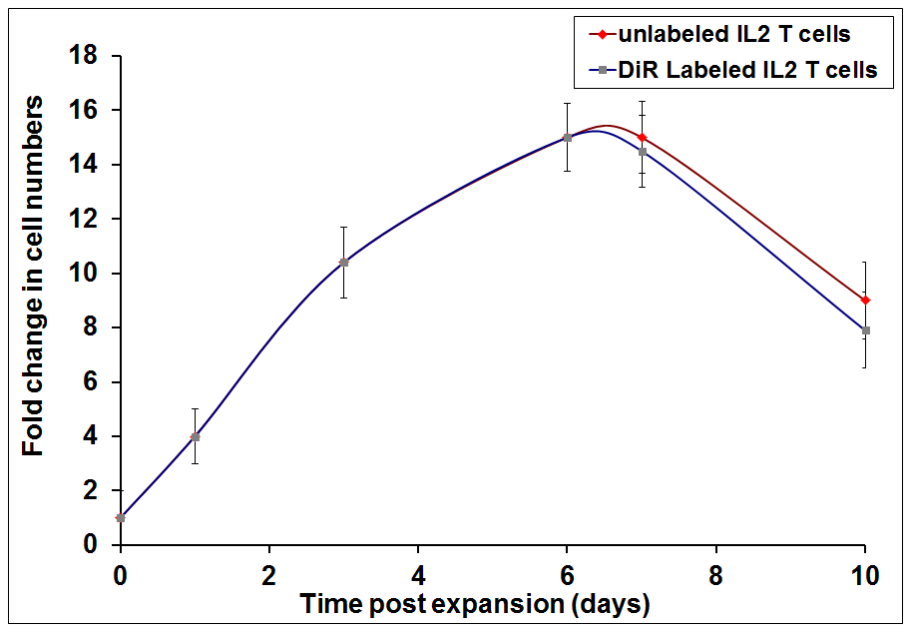
Fold increase in cell numbers measured by viable cell counts following *ex vivo* activation. Cell numbers were highest on day 6 and this time point was selected for DiR labeling. Following labeling, no significant differences (p<0.05) in cell proliferation between DiR labeled and unlabeled cells were found.

The proliferation of 4T1 sensitized lymphocytes isolated from tumor DLN activated with B/I and expanded in IL-2 containing media was observed for 13 days. Since the number of lymphocytes from day 6 was high (Figure 2), these were labeled with DiR to be adoptively transferred into 4T1 bearing mice, and the effect of labeling cells with DiR on proliferation was determined. The results confirmed that, cells labeled with DiR showed similar proliferation as the unlabeled T cells. Both unlabeled and labeled cells continue to proliferate at the same rate until day 4 post-labeling (day 10 of expansion). After that, the cell viability begins to go down (day 7 post-labeling which is day 13 of *ex vivo* expansion). These results showed that labeling of cells with DiR does not affect the proliferation of the lymphocytes grown in the presence of IL-2 even after 10 days of expansion.

### Interferon-γ production in vitro and in vivo

**Figure 3:**
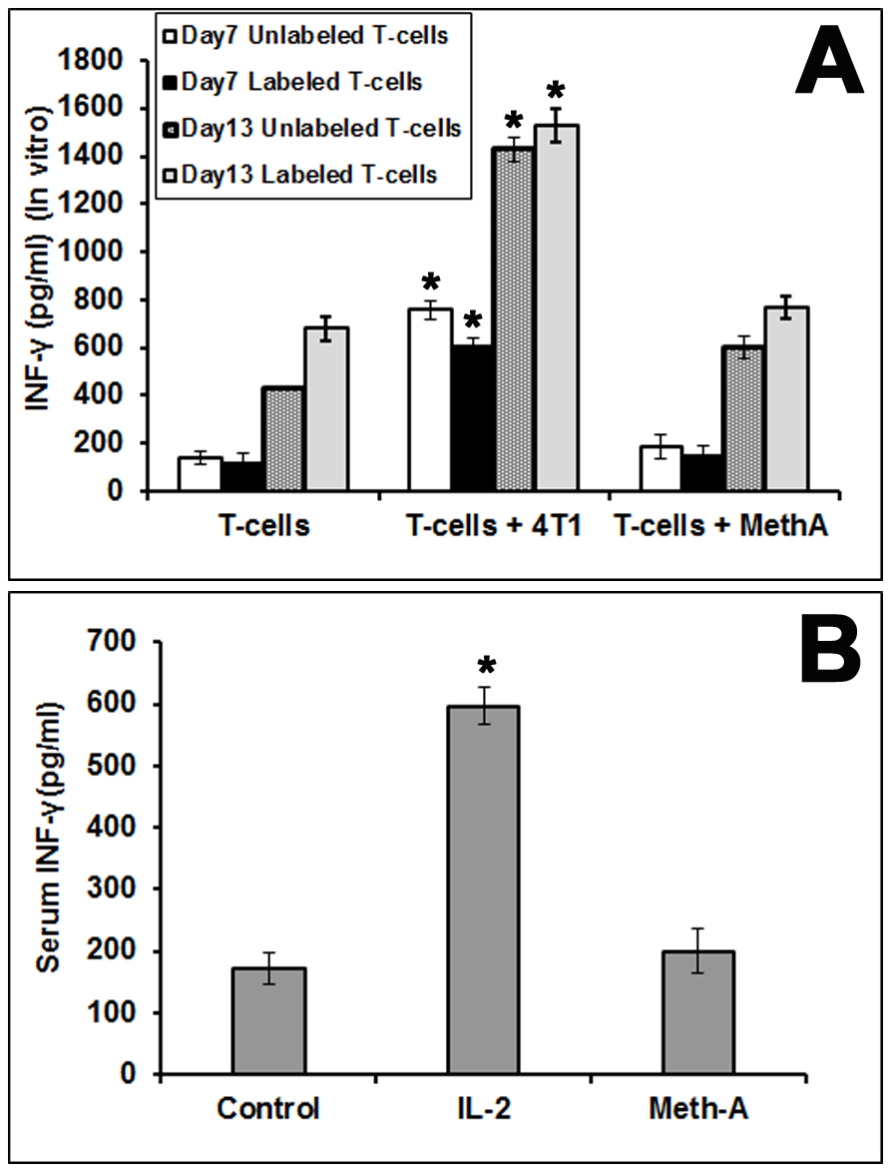
Interferon-gamma assay. The amount of interferon-gamma (IFN- γ) released into the supernatant (A) and in the mouse serum (B) by the 4T1 specific T lymphocytes are shown (mean ± SD). This demonstrates the 4T1 cell specificity and functioning of T lymphocytes are unaffected, both *in vitro* as well as *in vivo*, by the DiR labeling.

In vitro activated 4T1 sensitized T cells were expanded for 6 days, and incubated (either unlabeled or DiR-labeled) with irradiated 4T1 tumor cells or Meth A tumor cells for 1 and 7 days in IL-2 containing media. The levels of IFN-γ in the supernatant were determined. The results showed that, with time there is accumulation of IFN-γ in the media but there is no significant difference in the IFN-γ levels in the supernatant of T cells, incubated alone compared to T cells incubated with non-specific Meth A tumor (Figure 3 panel A) on respective days. However, T cells incubated with 4T1 tumor cells showed significantly higher release of IFN-γ into the media (p<0.05). Since no significant differences in cytokine levels released were observed between unlabeled and labeled T cells, the data also demonstrate that labeling the cells with DiR does not affect their interaction with tumor cells. Figure 3 panel B shows that, the amount of IFN-γ in the mice bearing 4T1 tumor which have been injected with labeled T cells, is higher in their blood serum compared with mice bearing 4T1 tumor only (no T cells infused) and mice bearing Meth A and which have been injected with labeled T cells.

### Multi-spectral fluorescent imaging of T cell trafficking

**Figure 4:**
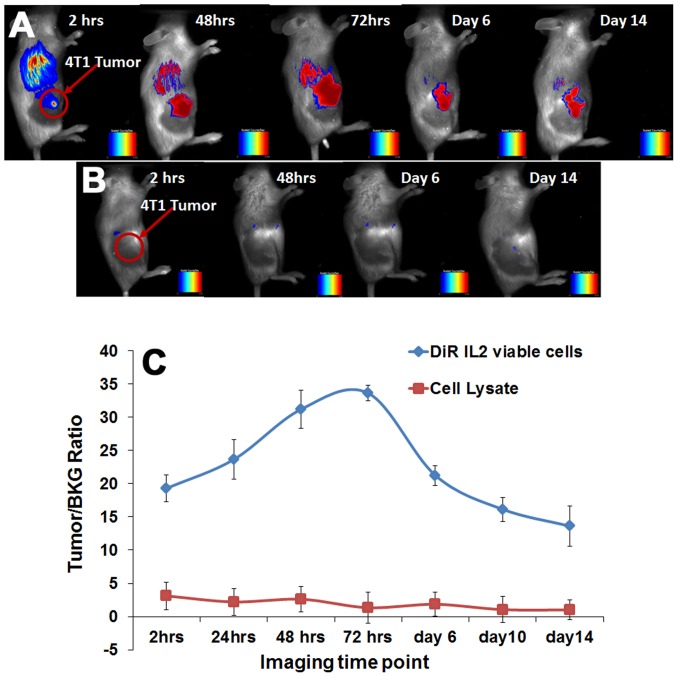
NIR fluorescence imaging of IL-2 grown T-cell trafficking. Homing of 4T1 sensitized DiR labeled viable cells to 4T1 tumor site was seen as early as 2 hours post T-cell administration (A). Fluorescent signal was not detected at the tumor site in animals injected with the cell lysates of DiR labeled IL-2 grown T-cells (B). Quantitation of Tumor/Background ratios show, T-cells localized at the tumor site from 2 hours posts T-cell administration, peaked on day 3 and persisted up to 14 days in the animal (C). While in case of IL-2 lysate, there was no localization of 4T1 specific T cells at the tumor site.

Adoptive transfer of DiR labeled 4T1 sensitized T lymphocytes which had been expanded in vitro for 6 days, were administrated intravenously after 10 days of the tumor implantation. Imaging of the mice using Maestro-2, revealed homing of 4T1 sensitized lymphocytes to the site of 4T1 tumor (Figure 4A). The signals from DiR labeled cells persisted in tumor bearing mice for up to 21 days (Figure S5A in File S1). The tumor/BKG ratio shows that the peak signal intensity is obtained on day 3 after adoptive transfer, following which the signal intensity started to gradually diminish (Figure 4C). In contrast, when the 4T1 sensitized lymphocytes were injected into Meth A tumor bearing mice, there was no migration of DiR labeled cells to the tumor site (Figure S5B & S5C in File S1). This is indicated by lack of fluorescence signal obtained from the tumor site. The absence NIR signal at the tumor site in mice injected with cell lysates confirms that only viable cells home into the tumor (Figure 4B).

**Figure 5:**
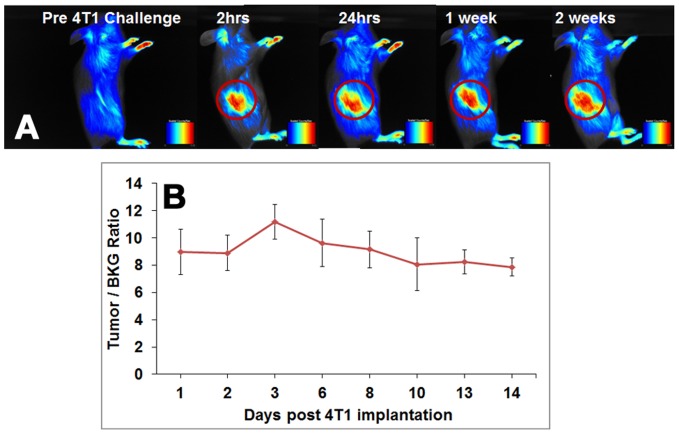
T-cell trafficking following delayed immunologic challenge. 4T1 sensitized T cells expanded in IL-2 containing media, were injected in naive mice one week prior to 4T1 challenge. (A) The labeled 4T1 specific T cells were able to leave the lymphoid compartments and localize within 2 hours at the site of an immunologic challenge induced by 4T1 cell implantation in the flank. (B) Tumor/Background ratios obtained from mice injected with T cells and implanted with 4T1 cells a week later.

To determine whether signals from DiR labeled 4T1 sensitized T cells can persist *in vivo* for a prolonged period of time and respond to a later immunologic challenge, 4T1 tumor cells were introduced one week after the mice had already received infusion of activated/expanded T cells. Interestingly, the T cells migrated to the site of tumor as early as 2 hours post injection of tumor cells and the signals from DiR tagged T cells was detectable for more than 2 weeks (Figure 5A). Further examination of the tumor/background (BKG) ratio revealed that the signal intensity had increased modestly on day 3 and then began to gradually decline (Figure 5B).

### Localization of labeled T-lymphocytes at non-tumor tissues

**Figure 6:**
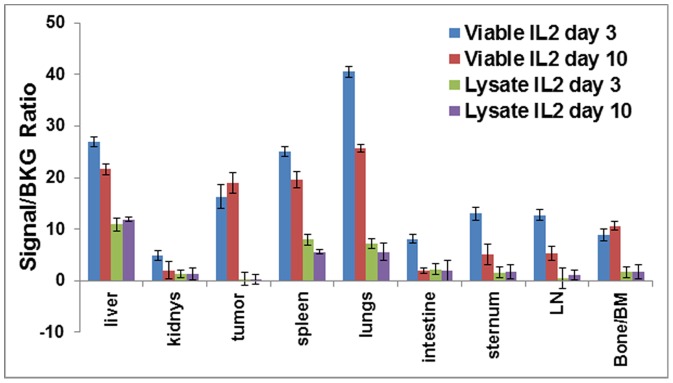
*Ex vivo* validation of mice injected with viable cells or T cell lysate. NIR signal from organs harvested from mice show 4T1 tumor specific accumulation of viable 4T1 sensitized T-lymphocytes, which is absent with T cell lysates.

To investigate the migration pattern of 4T1 sensitized T cells following inoculation to sites besides the tumor site, tumor bearing mice were dissected at various time points following inoculation with DiR labeled T cells and different organs were removed and imaged *ex vivo*. Besides tumor, labeled T lymphocytes were also seen to have localized to liver, spleen, lungs, and bone marrow, as shown in Figure 6. The maximum fluorescent signals from labeled T-lymphocytes in these organs were observed on days 7, 10 and 16 post-injection of labeled T-cells. The signal increased modestly with time, which would indicate that additional labeled cells were migrating to these sites or that the T-cells are proliferating over time at these organs.

### Immunohistochemistry confirmation of activated T cells at tumor site

**Figure 7:**
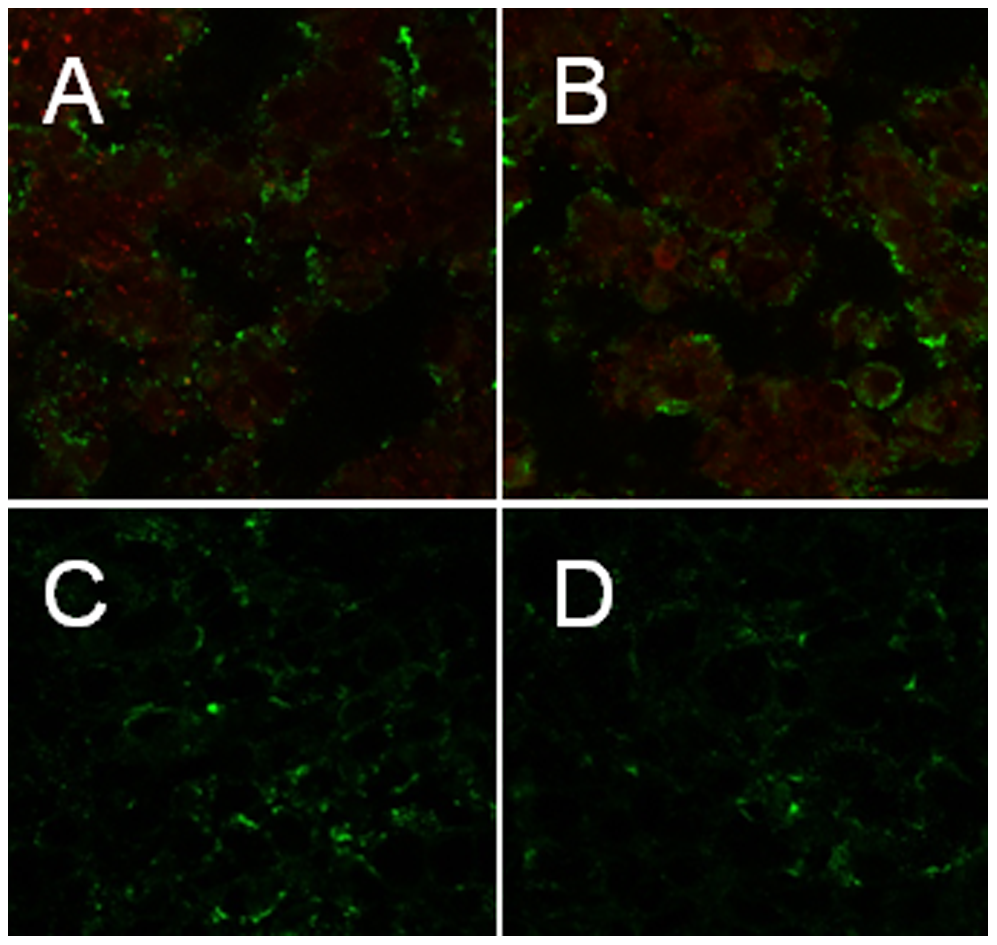
Histologic evaluation of T-cell accumulation in 4T1 tumors. 4T1 tumor bearing mice were injected with activated T-cells and the tumor sections were stained for CD69 activated T-cells (red color) marker and F4/80 macrophage (green color) marker (Figures-A & B). Images of 4T1 tumor sections obtained from mouse bearing 4T1 tumor but not injected with activated T cells, stained for CD69 and F4/80 markers (Figures-C & D).

To confirm the localization of the activated T-lymphocytes to the tumor site, 5 micron thick tumor sections from mice inoculated with DiR labeled T-cells were stained for CD69 and F4/80 to detect activated T-lymphocytes and macrophages respectively. Both activated T lymphocytes and macrophages were visualized at the tumor site as shown in Figure 7. Different sections of the tumor from outside to deep inside the tumor were analyzed. The activated T lymphocytes were mostly localized at the superficial layer of the tumor; macrophages were localized deeper inside the tumor tissues. To ensure that the localization of macrophages at the tumor site was not to attack and engulf labeled activated T-lymphocytes and behaves against these cells as foreign bodies, we implanted 4T1 breast carcinoma tumor in a number of mice and dissected them after 10 days when they have the same tumor size as in our previous experiments. These mice were not injected with activated T lymphocytes. Tumors were sectioned, double stained for CD69 and F4/80 and then imaged. Figure 7 C and D illustrate the images of these sections, and we did not see any activated T cells at these tumors and this was expected.

## Discussion

The present study demonstrates a simple optical imaging method to monitor T-lymphocytes that are sensitized to 4T1 murine breast carcinoma, and adoptively transferred to recipient mice bearing 4T1 tumors. Following transfer, tumor localization of the 4T1 specific T-lymphocytes started as early as 2 hours after intravenous administration and peaked at 3 days post administration of the activated cells. We also observed, from *ex vivo* imaging of dissected organs, that the labeled T-cells move into lymphoid organs such as bone marrow and spleen. Furthermore, we observed that when labeled 4T1 specific T cells were injected into non-tumor bearing mice, they migrated to 4T1 tumor cells that were implanted a week later. DiR labeled 4T1 specific T-cells were detected as early as 2 hours at the site of tumor cell injection. The signal at the tumor site persisted for another two weeks, confirming that the labeled T-cells can move out of the lymphoid organs to the site of a fresh antigen challenge. The use of DiR have been reported by other groups, where DiR was successfully used to stain human leukemia G2L cells, mouse lymphocytes and rat red blood cells for *in vivo* tracer experiments [36]. DiR was also used to label fibroblast cells, which were then observed to be targeting ovarian tumor some distance away from the injection site [37]. We are quite encouraged by the results of the present study in the breast cancer model. The DiR based direct cell membrane labeling might be a simple method that could help accelerate preclinical research in the area of immune cell homing to solid tumors. However, this method is not without some limitations, as discussed below.

A potential drawback of using a cell membrane dye is the possibility of dye transfer and cross labeling of neighboring cells both *in vitro* and *in vivo* [38]. Results from our initial *in vitro* experiments showed that such transfer of dye did not occur. This was confirmed by the lack of a fluorescent signal from 4T1 tumor cells, after these cells were incubated along with DiR labeled 4T1 sensitized T-cells for 24 hrs. (Figure S2 in File S1). Fluorescence detection of DiR in the liver, heart and 4T1 tumor obtained from mice injected with only the DiR dye revealed the presence of fluorescence only in the liver and not in the tumor or heart (Figure S4 in File S1) suggesting its retention in the liver for metabolism. Further, when lysates of DiR labeled cells are injected into 4T1 tumor bearing mice, NIR signal at the tumor site was absent. This observation suggested to us that the signal observed at the tumor site originated from the DiR labeled T-cells. These results and the results from *ex vivo* assays, including flowcytometry analysis which shows lack of significant DiR signal associated with macrophages and CD45^−^ non-immune cells (Figure S3 in File S1), confirm that the NIR signal emanating from the tumor site pertains to DiR labeled activated T-cells. Thus trafficking of tumor specific T-cells can be non-invasively monitored by the direct labeling of T-cells with DiR. Adding further support are reports by other investigators who have also used similar techniques to monitor cellular therapy without any hindrance from non-specific transfer of the dye [39]. For the applications described in the present study, the small degree of dye transfer that may occur toward the neighboring cells does not appear to be a limiting issue.

Having demonstrated the utility of this imaging method to show localization and persistence of labeled T-cells at the 4T1 tumor sites, we wanted to verify that the T cells retained at the tumor do actually inhibit 4T1 tumor growth. Our data (Figure S6 in File S1) show significant inhibition of 4T1 tumors into which labeled T-cells migrated, similar to unlabeled 4T1 specific T-cells. Furthermore we show that this inhibition is specific to 4T1 tumors because we demonstrate that inhibition of tumor growth was not observed in Meth-A tumor, to which 4T1 T-cells are not specific. Similar findings were observed by Cha and coworkers, who showed that 4T1 specific T cells grown in IL-2 media are able to inhibit 4T1 tumor growth [40].

Tracking of cells using genetically encoded markers has its own shortcomings. Currently, PET reporter gene imaging of transduced cells appears to be best suited for *in vivo* and clinical applications. Herpes simplex virus type 1 thymidine kinase (HSV1-tk) and its mutant HSV1-sr39tk are the most widely used PET reporter genes and have been extensively studied for cell and gene therapy [41]. However, PET imaging of reporter genes involves the administration of a radioactive probe that is selectively trapped inside the cells. Since HSV1-tk is a protein of viral origin, in humans, immune reactions against it are a serious concern for its use in transplanted cells that are intended to survive for longer periods [42], [43]. Furthermore, currently available reporter probes for HSV1-tk or HSV1-sr39tk are also phosphorylated, to a small degree, by mammalian thymidine kinases. Therefore, some non-specific uptake of these reporter probes are seen in rapidly proliferating mammalian cells, such as cancer cells, which will limit the detection of lymphocytes expressing HSV1-sr39tk within the tumor tissue [24]. Currently available radiolabeled probes for PET reporter genes have unfavorable biologic clearance properties (high background in the abdominal region) or have other physical properties (non-pure positron-emitting isotope and/or long physical half-life of the radiolabel) that are not ideal for PET imaging [42]. Transgene delivery to dividing and non-dividing cells is not easy. Despite several advances in transgene delivery methods, the transduction of primary human T cells is still challenging and methods to achieve efficient gene transfer are often expensive and time-consuming. Considering the above, it is evident that gene based cell imaging techniques are also not without limitations.

Smirnov and colleagues [44], studied *in vivo* T cell trafficking by direct labeling with SPIONs for MRI imaging. Their results showed relatively lower signal in the spleen at 24 hours than at 48 hours. The signal in spleen and tumor then peaked at 72 hours. In another study done by Pittet and colleagues [45], it was shown that tumor specific cytotoxic T-lymphocytes directly labeled with ^111^In-Oxine (2.8 days half-life) for SPECT imaging, started localizing at the tumor site within the first 24 hours and continued to increase over time and were at peak when imaged at 120 hours post injection. Labeling of T-cells with near-infra red (NIR) fluorescent dyes for AIT has been reported in a few recently published studies. Du and coworkers [39], investigated the trafficking of DiR labeled human cytokine-induced killer (CIK) cells and cytotoxic T lymphocytes (CTLs) in a nude mouse model of orthotopic gastric carcinoma. In their study, the fluorescent signal peaked at 48 hours and concentrated fluorescence signals was observed at the tumor site, up to two weeks after infusion. Our results are consistent with these reports; and moreover, the NIR signal from the T-cells at the tumor site persisted for nearly three weeks after labeling and administration. This also demonstrates the suitability of this method for relatively long-term studies.

After labeling the T-cells with a range of DiR concentrations, we tested the cell viability using two different methods to ensure good cell tolerance to the dye before moving to the *in vivo* studies. T-cell function was also tested by measuring IFN-γ release in response to tumor antigen, and our results showed no significant detrimental effect of the labeling probe on cell proliferation, viability or function. Also, this allowed us to use a high DiR concentration (320 µg/ml) in the labeling solution. Our study also demonstrates persistence of signal associated with labeled T-cells even 3 weeks post labeling under the *in vivo* conditions of living mice. In the study by Du and coworkers [39], the cell proliferation of labeled cells was comparable to unlabeled cells, similar to our observations.

The *in vivo* biodistribution of the labeled T-lymphocytes, in this study, was consistent with other studies. The NIR signal was detected in lungs, spleen, liver, bone marrow and was observed at the tumor site at 24 hours and remained stable for up to 21 days when the labeled cells were infused into mice bearing established 4T1 breast carcinoma tumors. To ascertain whether the source of signal around the tumor was coming from labeled T-cells, double staining of tumor with CD69 (T cell activation marker) and F4/80 (macrophage marker), was performed. The confocal microscopy images revealed presence of activated T cells only in sections from mice bearing 4T1 tumor injected with *ex vivo* activated T cells and not in tumor sections from control 4T1 bearing mice. On the other hand, the presence of macrophages was observed in tumor sections from both groups of animals as expected. However, it is quite possible that a portion of the signal from lungs, spleen, liver and bone marrow could emanate from T-cells that were labeled with DiR, but died at some point following *in vivo* administration. Because a portion of the DiR labeled cell lysate was observed to be accumulating in these organs. This could impede the interpretation of images if the targeted tumors are located in any of these organs. However, it should be noted that the signal coming from the viable cells three days post injection are at least one fold greater than the signal coming from the dead cells. Such intense signal at the tumor site could serve as a clue to distinguish tumor specific localization from non-specific accumulation.

In conclusion, this study shows that, labeling of 4T1 specific T lymphocytes with DiR, a NIR fluorescent cell membrane marker, provides an easy to use, relatively stable and convenient tool for *in vivo* imaging of T-lymphocytes whilst retaining their inherent function. Use of DiR or similar cell membrane markers will be useful for investigating lymphocyte trafficking following their *ex vivo* sensitization, activation and proliferation. This method could also be used to non-invasively monitor the trafficking of other cell types. We are encouraged by our findings and recommend the use of this technique by other investigators, to further evaluate and utilize this strategy. When appropriate validations are included, this method could be easily adopted for preclinical research without the need of sophisticated radionuclide imaging facilities.

## Supporting Information

File S1 **This file contains Figure S1–Figure S6.** Figure S1: DiR labeled T cells were analyzed by flowcytometry showing the mean fluorescent intensity (MFI) is markedly high with DiR labeled CD3 positive T cells and the labeling efficiency with DiR was 94 %. Figure S2: DiR labels T cells with negligible transfer to tumor cells. DiR labeled T cells incubated with 4T1 tumor cells for 24 hrs, showed negligible to no transfer of dye to 4T1 cells. Figure S3: Confirmation of immunohistochemistry results by flowcytometry. Panel-A tissues were stained for macrophages (F4/80), panel-B tissues were stained for immune cell marker CD45. Both confirm that DiR is linked to T cells and not transferred to other cells. Figure S4: Unmixed fluorescent images of liver, heart and 4T1 tumor sections of animal injected with DiR only. From the ex vivo imaging higher signal was detected in the liver and no signal was in the heart, that is why liver was used as positive and heart negative controls. 4T1 tumor section showed no signal of DiR indicating that, the signal detected at the tumor site is correlated to DiR labeled cells. Figure S5: Fluorescence imaging of T cell trafficking. Homing of 4T1 sensitized DiR labeled T cells to (Panel A) 4T1 tumor site (green color codes for Autofluorescence signal, and red color codes for DiR signal), (Panel B) Meth-A carcinoma tumor site (used as negative control tumor) 4 days after the tumors have been implanted. Red color indicates the signal from the NIR DiR Dye used to label the T cells. (C) Tumor/Background ratios graph showed that, cells localized at the tumor site on day 1 peaked on day 6 and persisted up to 21 days in the animal. While in case of Meth A tumor, there was no localization of 4T1 specific T cells at the tumor site. Figure S6: T cells with and without DiR labeling inhibit 4T1 tumor growth in mice (n = 6/group). (A) Untreated control (Ctrl 4T1) and Cyclophosphamide (CYP) only groups (CYP 4T1) showed increase in the 4T1 tumor volumes over time, while mice treated with CYP and 4T1 sensitized T cells (CYP AIT 4T1) inhibited 4T1 tumor growth. This function was unaffected by DiR labeling of the 4T1 sensitized T cells (CYP AIT 4T1 DiR). (B) The specificity of the 4T1 sensitized T cells against 4T1 tumor is demonstrated by the absence of tumor growth inhibition when these T cells were used against Meth-A tumors in mice.
(PDF)
