# Supplementary material for: Near-Infrared Imaging of Adoptive Immune Cell Therapy in Breast Cancer Model Using Cell Membrane Labeling
Source: PLoS One. 2014 Oct 21;9(10):e109162. doi: 10.1371/journal.pone.0109162 (PMC4204826; doi:10.1371/journal.pone.0109162)
Supplement: File S1 — This file contains Figure S1–Figure S6. Figure S1: DiR labeled T cells were analyzed by flowcytometry showing the mean fluorescent intensity (MFI) is markedly high with DiR labeled CD3 positive T cells and the labeling efficiency with DiR was 94 %. Figure S2: DiR labels T cells with negligible transfer to tumor cells. DiR labeled T cells incubated with 4T1 tumor cells for 24 hrs, showed negligible to no transfer of dye to 4T1 cells. Figure S3: Confirmation of immunohistochemistry results by flowcytometry. Panel-A tissues were stained for macrophages (F4/80), panel-B tissues were stained for immune cell marker CD45. Both confirm that DiR is linked to T cells and not transferred to other cells. Figure S4: Unmixed fluorescent images of liver, heart and 4T1 tumor sections of animal injected with DiR only. From the ex vivo imaging higher signal was detected in the liver and no signal was in the heart, that is why liver was used as positive and heart negative controls. 4T1 tumor section showed no signal of DiR indicating that, the signal detected at the tumor site is correlated to DiR labeled cells. Figure S5: Fluorescence imaging of T cell trafficking. Homing of 4T1 sensitized DiR labeled T cells to (Panel A) 4T1 tumor site (green color codes for Autofluorescence signal, and red color codes for DiR signal), (Panel B) Meth-A carcinoma tumor site (used as negative control tumor) 4 days after the tumors have been implanted. Red color indicates the signal from the NIR DiR Dye used to label the T cells. (C) Tumor/Background ratios graph showed that, cells localized at the tumor site on day 1 peaked on day 6 and persisted up to 21 days in the animal. While in case of Meth A tumor, there was no localization of 4T1 specific T cells at the tumor site. Figure S6: T cells with and without DiR labeling inhibit 4T1 tumor growth in mice (n = 6/group). (A) Untreated control (Ctrl 4T1) and Cyclophosphamide (CYP) only groups (CYP 4T1) showed increase in the 4T1 tumor volumes over time, w [file pone.0109162.s001.pdf]

SUPPORTING INFORMATION

Near-Infrared Imaging of Adoptive Immune Cell Therapy in Breast Cancer Model using Cell Membrane Labeling

Fatma M. Youniss[1], Gobalakrishnan Sundaresan[1], Laura J. Graham[2], Li Wang[1], Collin R. Berry[1], Gajanan K. Dewkar[1], Purnima Jose[1], Harry D. Bear[2,3], Jamal Zweit,[1,3]*

Affiliations:

[1]Department of Radiology, Center for Molecular Imaging, Virginia Commonwealth University, Richmond, Virginia, USA

[2]Department of Surgery, Division of Surgical Oncology, Virginia Commonwealth University, Richmond, Virginia, USA

[3]Massey Cancer Center, Virginia Commonwealth University, Richmond, Virginia, USA

Supplementary Figures (6 figures)

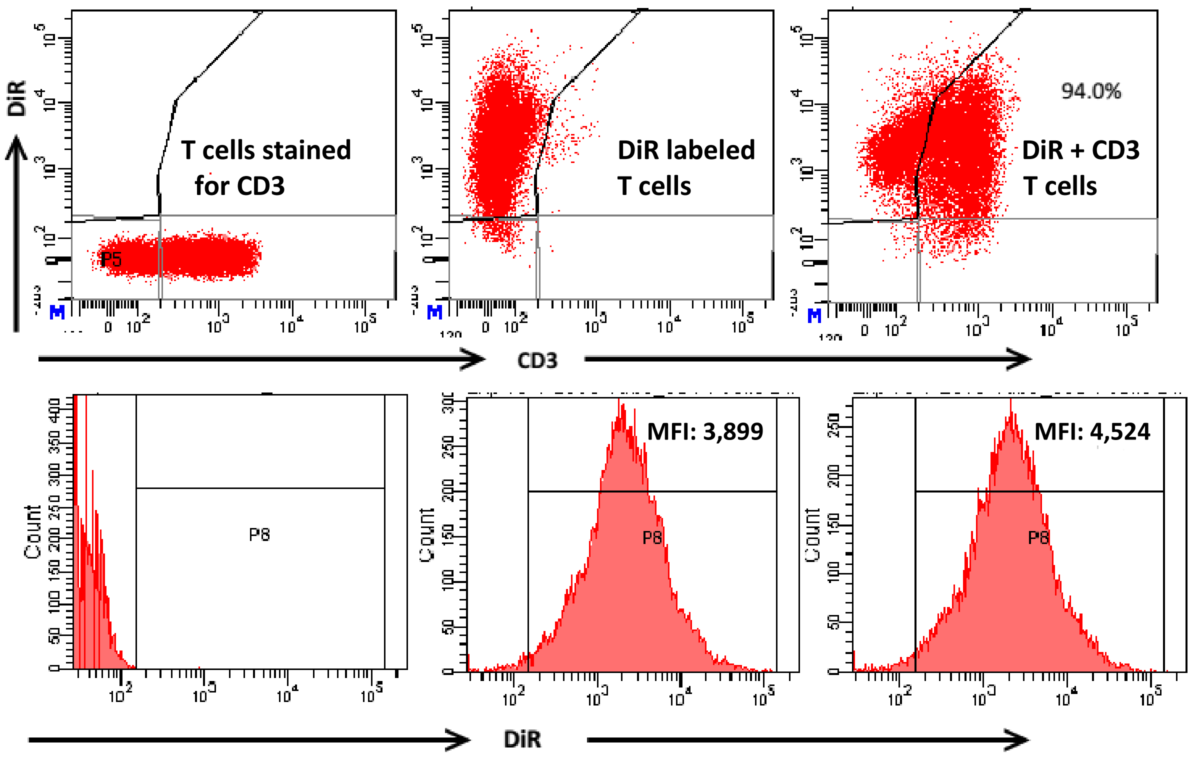

Figure S1: DiR labeling efficiency measured by flow cytometry. DiR labeled T cells were analyzed by flowcytometry showing the mean fluorescent intensity (MFI) is markedly high with DiR labeled CD3 positive T cells and the labeling efficiency with DiR was 94 %.

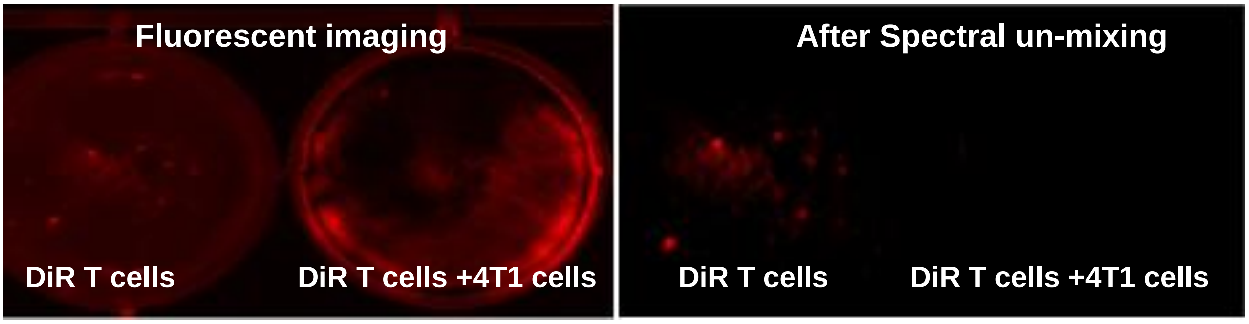

Figure S2: DiR labels T cells with negligible transfer to tumor cells. DiR labeled T cells incubated with 4T1 tumor cells for 24 hrs, showed negligible to no transfer of dye to 4T1 cells.

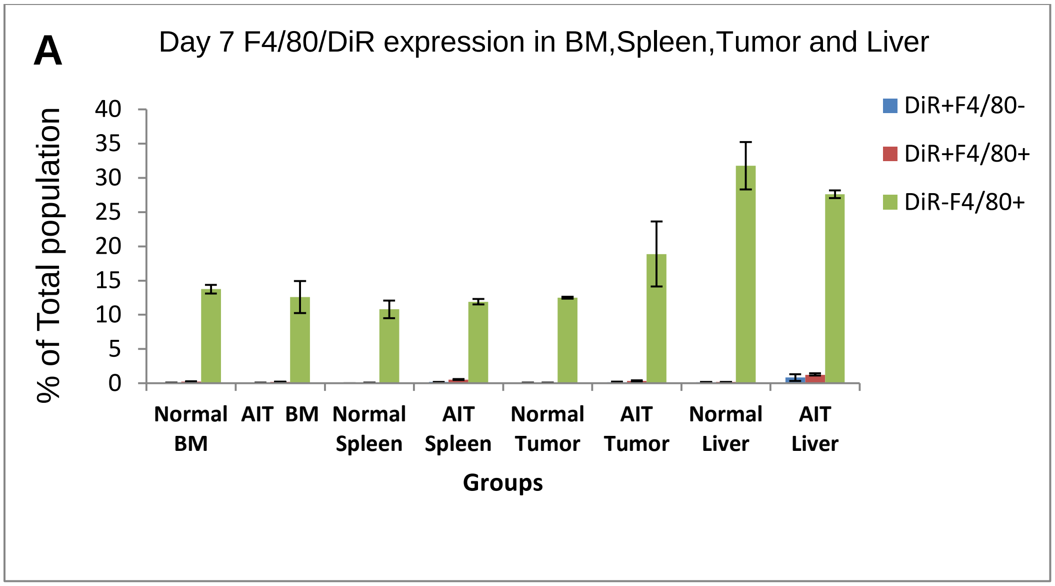

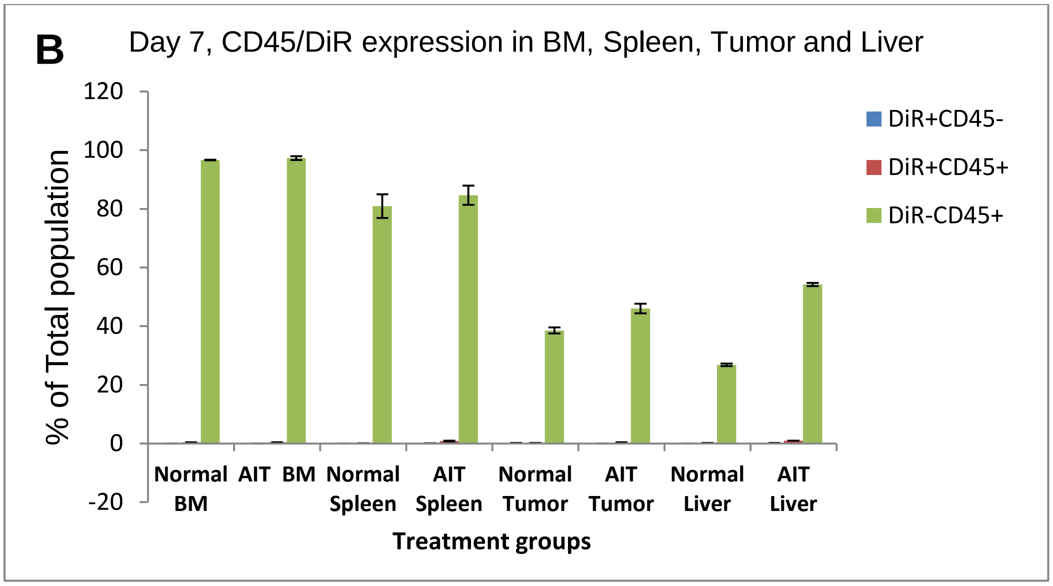

Figure S3: Confirmation of immunohistochemistry results by flowcytometry. P Cells were obtained after enzymatic digestion of tissues from Normal mice and AIT mice. Panel-A tissues were stained for macrophages (F4/80). Panel-B tissues were stained for immune cell marker CD45. Both confirm that DiR is linked to T cells and not transferred to other cells. AIT = Adoptive Immune Therapy; BM = Bone marrow.

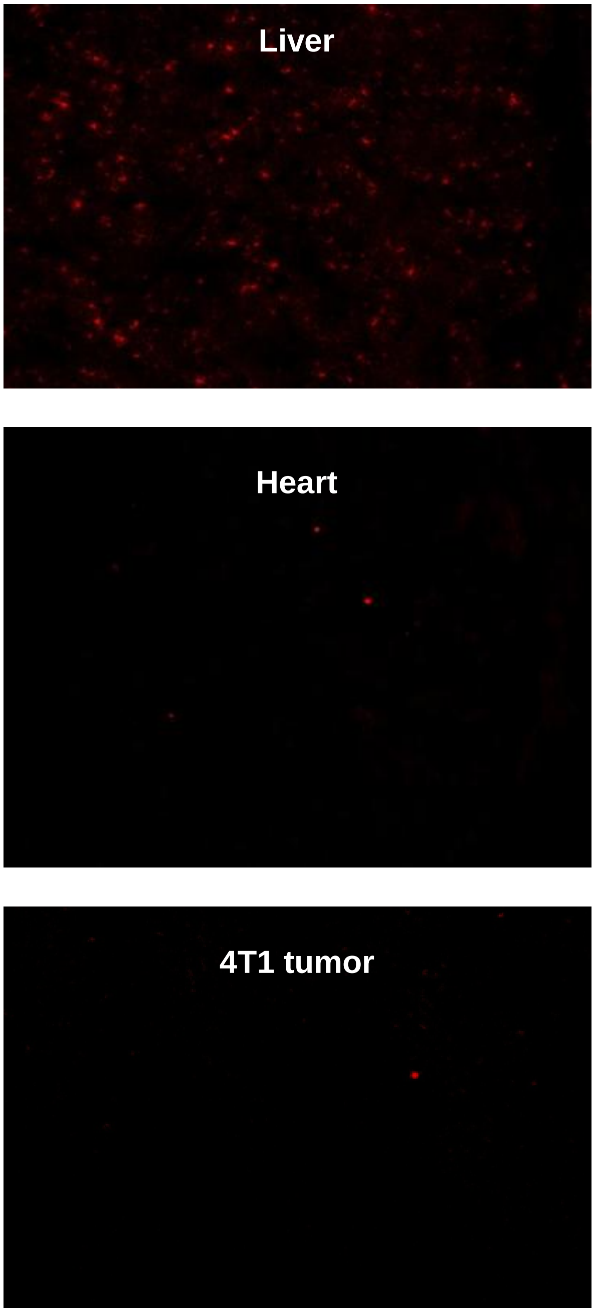

Figure S4: Unmixed fluorescent images of liver, heart and 4T1 tumor sections of animal injected with DiR only. From the ex vivo imaging higher signal was detected in the liver and no signal was in the heart, that is why liver was used as positive and heart negative controls. 4T1 tumor section showed no signal of DiR indicating that, the signal detected at the tumor site is correlated to DiR labeled cells.

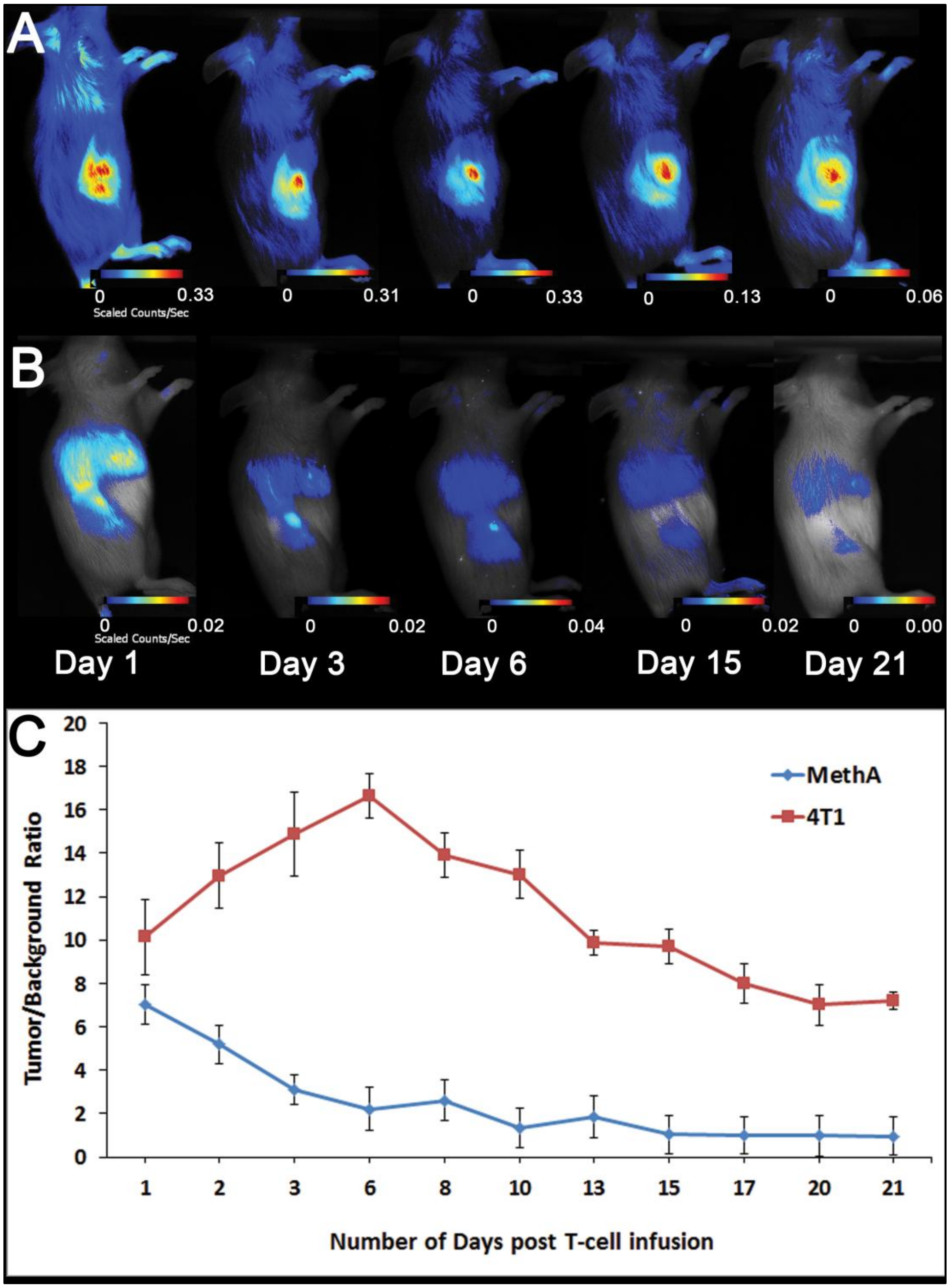

Figure S5: Fluorescence imaging of T cell trafficking. Homing of 4T1 sensitized DiR labeled T cells to (Panel A) 4T1 tumor site (green color codes for Autofluorescence signal , and red color codes for DiR signal), (Panel **B)** Meth-A carcinoma tumor site (used as negative control tumor) 4 days after the tumors have been implanted. Red color indicates the signal from the NIR DiR Dye used to label the T cells. (**C)** Tumor/ Background ratios graph showed that, cells localized at the tumor site on day 1 peaked on day 6 and persisted up to 21 days in the animal. While in case of Meth A tumor, there was no localization of 4T1 specific T cells at the tumor site.

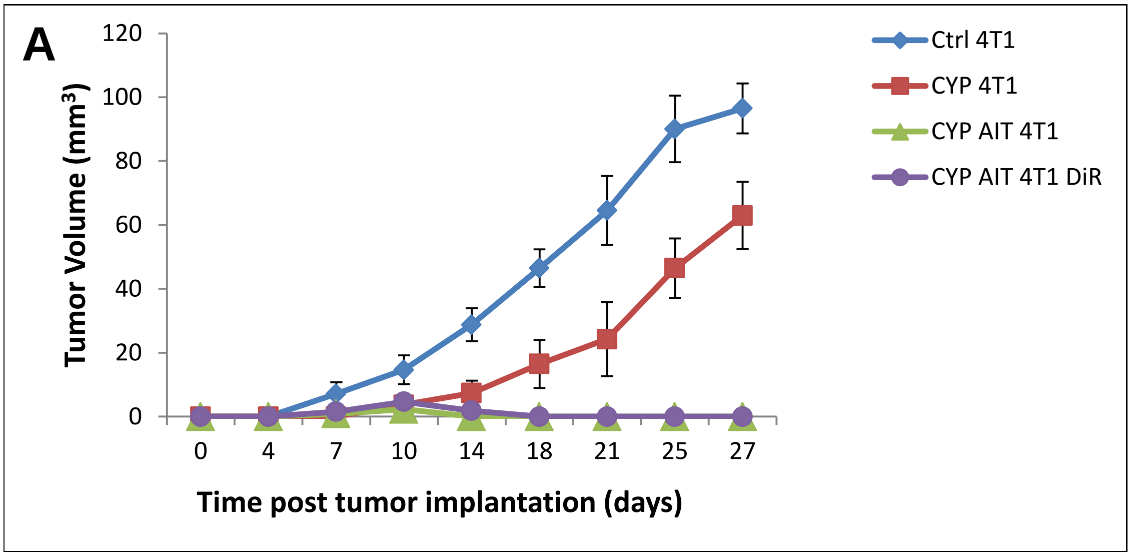

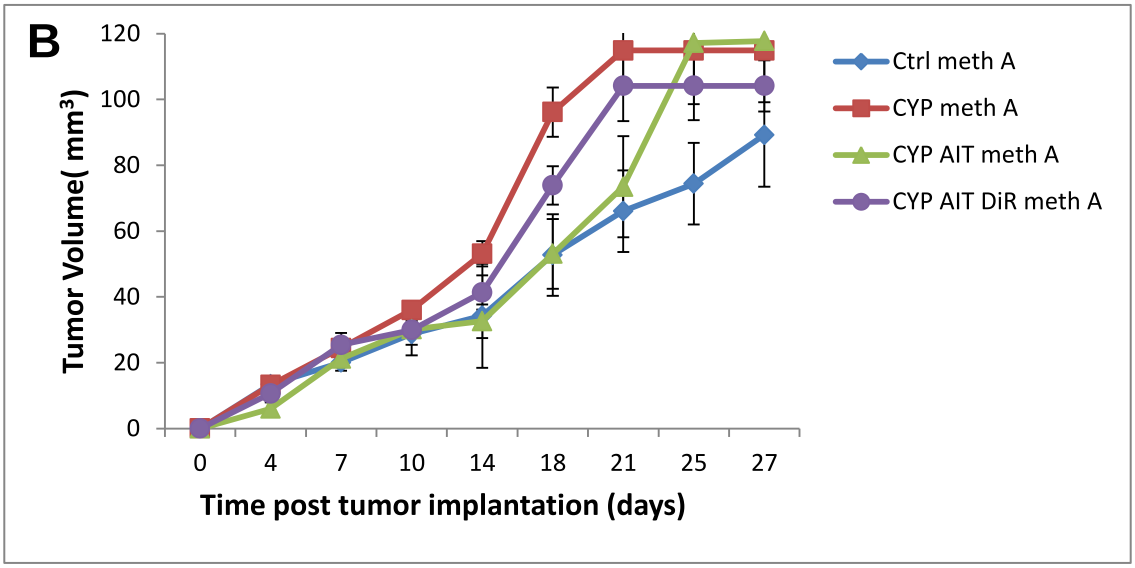

Figure S6: Effect of DiR labeling 4T1 tumor growth. T cells with and without DiR labeling inhibit 4T1 tumor growth in mice (n=6/group). (**A**) Untreated control (Ctrl 4T1) and Cyclophosphamide (CYP) only groups (CYP 4T1) showed increase in the 4T1 tumor volumes over time, while mice treated with CYP and 4T1 sensitized T cells (CYP AIT 4T1) inhibited 4T1 tumor growth. This function was unaffected by DiR labeling of the 4T1 sensitized T cells (CYP AIT 4T1 DiR). (**B**) The specificity of the 4T1 sensitized T cells against 4T1 tumor is demonstrated by the absence of tumor growth inhibition when these T cells were used against Meth-A tumors in mice.
